# Spaced Digital Education for Health Professionals: Systematic Review and Meta-Analysis

**DOI:** 10.2196/57760

**Published:** 2024-10-10

**Authors:** Laura Martinengo, Matthew Song Peng Ng, Tony De Rong Ng, Yi-Ian Ang, Ahmad Ishqi Jabir, Bhone Myint Kyaw, Lorainne Tudor Car

**Affiliations:** 1 Lee Kong Chian School of Medicine Nanyang Technological University Singapore Singapore Singapore; 2 Centre for Behavioural and Implementation Sciences Interventions Yong Loo Lin School of Medicine National University of Singapore Singapore Singapore; 3 Yong Loo Lin School of Medicine National University of Singapore Singapore Singapore; 4 Future Health Technologies Singapore-ETH Centre Campus for Research Excellence And Technological Enterprise Singapore Singapore; 5 Nanyang Technological University Singapore Singapore Singapore; 6 Department of Primary Care and Public Health School of Public Health Imperial College London London United Kingdom

**Keywords:** digital education, e-learning, spaced education, spaced learning, spaced digital education, spaced simulation, health care professionals, continuous medical education, systematic review, meta-analysis

## Abstract

**Background:**

Spaced digital education applies digital tools to deliver educational content via multiple, repeated learning sessions separated by prespecified time intervals. Spaced digital education appears to promote acquisition and long-term retention of knowledge, skills, and change in clinical behavior.

**Objective:**

The aim of this review was to assess the effectiveness of spaced digital education in improving pre- and postregistration health care professionals’ knowledge, skills, attitudes, satisfaction, and change in clinical behavior.

**Methods:**

This review followed Cochrane’s methodology and PRISMA (Preferred Reporting Items of Systematic Reviews and Meta-Analyses) reporting guidelines. We searched MEDLINE, Embase, Web of Science, ERIC, PsycINFO, CINAHL, CENTRAL, and ProQuest Dissertation and Theses databases from January 1990 to February 2023. We included randomized controlled trials (RCTs), cluster RCTs, and quasi-RCTs comparing spaced digital education with nonspaced education, spaced nondigital education, traditional learning, or no intervention for pre- or postregistration health care professionals. Study selection, data extraction, study quality, and certainty of evidence were assessed by 2 independent reviewers. Meta-analyses were conducted using random effect models.

**Results:**

We included 23 studies evaluating spaced online education (n=17, 74%) or spaced digital simulation (n=6, 26%) interventions. Most studies assessed 1 or 2 outcomes, including knowledge (n=15, 65%), skills (n=9, 39%), attitudes (n=8, 35%), clinical behavior change (n=8, 35%), and satisfaction (n=7, 30%). Most studies had an unclear or a high risk of bias (n=19, 83%). Spaced online education was superior to massed online education for postintervention knowledge (n=9, 39%; standardized mean difference [SMD] 0.32, 95% CI 0.13-0.51, *I*^2^=66%, moderate certainty of evidence). Spaced online education (n=3, 13%) was superior to massed online education (n=2, 9%) and no intervention (n=1, 4%; SMD 0.67, 95% CI 0.43-0.91, *I*^2^=5%, moderate certainty of evidence) for postintervention clinical behavior change. Spaced digital simulation was superior to massed simulation for postintervention surgical skills (n=2, 9%; SMD 1.15, 95% CI 0.34-1.96, *I*^2^=74%, low certainty of evidence). Spaced digital education positively impacted confidence and satisfaction with the intervention.

**Conclusions:**

Spaced digital education is effective in improving knowledge, particularly in substantially improving surgical skills and promoting clinical behavior change in pre- and postregistration health care professionals. Our findings support the use of spaced digital education interventions in undergraduate and postgraduate health profession education. Trial Registration: PROSPERO CRD42021241969;

**Trial Registration:**

PROSPERO CRD42021241969; https://www.crd.york.ac.uk/prospero/display_record.php?RecordID=241969

## Introduction

### Background

Health care service delivery depends on access to a competent workforce. The World Health Organization (WHO) has long warned of severe global shortages of health care workers, particularly in low- and middle-income countries [1,2]. Long working hours, low wages, workplace stress, and lack of professional development opportunities [3,4] appear to drive the health care workforce turnover [3]. An important strategy to increase recruitment and retention of health care workers is providing high-quality education in an affordable, effective, and sustainable way [5].

Digital education is “the act of teaching and learning by means of digital technologies” [6]. Digital education may promote continuous professional development and improve health care workers’ competencies by offering convenient and adaptable learning tools that can be accessed at any place and time [6-10]. A recent series of systematic reviews and meta-analyses on different digital education modalities [6,11-18] showed that digital education is well received and noninferior to traditional education.

### Spaced Education

Spaced education is an educational technique that promotes long-term knowledge retention via multiple repeated learning sessions separated by prespecified time intervals [19-21]. Spacing knowledge acquisition is a well-studied and effective learning technique, particularly if it is associated with the active retrieval of information [22]. The beneficial effects of spacing and retrieval appear to be independent of the age of students and the length of the spacing interval, although research seems to suggest that longer spacing intervals are correlated to longer-term knowledge retention [22,23]. Similarly, the effectiveness of spaced education appears to be comparable in online, simulation, and classroom settings [24] or in studies assessing the acquisition of theoretical knowledge and practical skills [22,24]. Spaced education has been increasingly used in undergraduate [25] and postgraduate [24] medical education to improve learners’ knowledge [26] or skills, such as resuscitation in emergency medicine [27,28], surgical techniques [29], and image interpretation in radiology [30]. Spaced education has also been shown to promote changes in clinical practice [31,32]. By combining the accessibility and flexibility of digital education with the benefits of spaced learning in knowledge retention, spaced digital education might offer a feasible, scalable, and effective solution to providing high-quality education for health care professionals [33].

A recent systematic review [31] assessed the effectiveness of spaced education in continuous development programs for practicing health care professionals, suggesting an improvement in knowledge, skills, clinical behavior, and confidence. However, the review included a variety of research designs and offered a narrative summary of findings. Given the increasing relevance of spaced education in health profession education, particularly when delivered in digital format, we considered it important to update and expand Phillips et al’s [31] systematic review. The aim of this systematic review was to assess the effectiveness of spaced digital education in improving pre- and postregistration health care professionals’ knowledge, skills, attitudes, satisfaction, and clinical behavior change.

## Methods

### Study Design

The review followed Cochrane’s methodology [34] and was reported according to the PRISMA (Preferred Reporting Items of Systematic Reviews and Meta-Analyses) checklist [35]. The protocol was registered on the International Prospective Register of Systematic Reviews (PROSPERO; CRD42021241969) on April 14, 2021.

### Identification of Studies

The search strategy was developed for a series of systematic reviews on the effectiveness of digital health education interventions for the education and training of students and professionals of health care–related professions [5,12-14,17,18,36-47]. The search strategy was developed in collaboration with librarians and information specialists from Karolinska Instituet and has been regularly updated. We searched MEDLINE, CENTRAL, Embase, Web of Science, ERIC, PsycINFO, CINAHL, and ProQuest Dissertation and Theses databases from January 1990 to February 2023. We complemented the search with forward and backward citation searches in all included studies and 2 relevant reviews [24,31]. Multimedia Appendix 1 presents the MEDLINE search strategy.

### Eligibility Criteria

This systematic review included randomized controlled trials (RCTs), cluster randomized controlled trials (cRCTs), and quasi-RCTs reporting spaced digital education interventions for health profession education. Randomized cross-over trials were excluded due to the high risk of contamination and carryover effects in educational interventions.

Study participants included preregistration students and postregistration professionals from health professions, such as medicine, dentistry, nursing and midwifery, medical diagnostic and treatment technology, physiotherapy and rehabilitation, and pharmacy, as defined by in the Health Field of Education and Training (091) of the International Standard Classification of Education (ISCED-F) [48]. Practitioners of traditional, alternative, and complementary medicine were excluded.

We included studies comparing spaced digital education interventions with nonspaced digital education modalities, spaced nondigital education, traditional face-to-face learning, blended learning, or no intervention. We included 2 spaced digital education modalities in this review. Spaced online education was defined for this study as an educational intervention where information, such as case studies or multiple-choice questions, “is presented and repeated over spaced intervals” [24,49] using a digital device, such as a computer, smartphone, or tablet computer. Spaced digital simulation was defined as studies using high-fidelity simulators, including virtual reality (VR)–based instruments, to provide skills training and assessment that was repeated over spaced intervals. There may be 2 or more repetitions of the learning content that should occur in different days.

Studies were excluded if both intervention and comparison groups described a spaced digital education intervention, if the spacing interval was shorter than 1 day, and if the materials were not repeated over the course of the intervention.

### Outcomes Measured

We assessed the following *primary outcomes*, measured using validated or nonvalidated instruments:

- Postintervention knowledge
- Postintervention skills
- Postintervention attitudes or perceptions toward the intervention, as well as the interaction with patients and colleagues
- Learners’ satisfaction with the intervention
- Postintervention behavior change in the way learners interact with patients or colleagues or modify their practices

We also included the following *secondary outcomes*: patient-related outcomes, cost and cost-effectiveness of the intervention, and negative effects of implementing a spaced education intervention.

### Data Collection and Analysis

#### Selection of Studies

The title and abstract screening for previous updates of the library was performed by pairs of reviewers working independently and in parallel. However, for the February 2023 update of the search strategy, screening was performed by 1 reviewer (author LM) on ASReview [50,51], an open source screening tool using several active learning and machine learning techniques. Following ASReview guidelines, we defined stopping criteria of 1.04% (90/8634 citations) consecutive “irrelevant” records and screening between 8% and 33% of all citations. The stopping criterion for this review was reached after screening 2850 (33.01%) records. Full-text screening for the entire digital health education library was performed by groups of 2 independent reviewers (authors MSPN, TDRN, and YIA) working in parallel. The data extraction and quality assessment of the included studies were also performed by groups of 2 independent reviewers. Differences between reviewers were resolved by discussion or by consultation with a third reviewer (LM) acting as an arbiter.

#### Data Extraction and Quality Assessment

Data from the included studies were extracted using a standardized Microsoft Excel form. We extracted information about the study design, participants, characteristics of the digital health education intervention, primary and secondary outcomes, and pre- and postintervention results, as reported by the primary studies.

We assessed the methodological quality of the included studies using Cochrane’s Risk of Bias Tool 2 (RoB-2) [52,53], which assesses the risk of bias arising from the randomization process, deviations from the intended interventions, missing outcome data, measurement of the outcome, and selection of the outcome result. For cRCTs, we also assessed the bias arising from the timing of identification or recruitment of participants into clusters. Studies were assessed as having a high risk of bias if at least 1 category was assessed as high risk or multiple domains were assessed as “some concerns.” Studies with 1 domain assessed as “some concerns” were categorized as “some concerns” risk of bias. Finally, studies with all domains assessed as low risk were classified as having a low risk of bias. The risk-of-bias figures were created using robvis, an online tool [54].

#### Grading the Quality of the Evidence

We assessed the quality of evidence and presented it as a summary-of-findings table (Multimedia Appendix 2) for 3 comparisons: spaced online education versus no intervention, spaced online education versus massed online education, and spaced digital simulation versus massed simulation. The evaluation followed the Grading of Recommendations, Assessment, Development and Evaluation (GRADE) criteria; assessed the limitation of the studies (risk of bias), inconsistency, indirectness, and imprecision; and rated the quality of evidence as very low, low, moderate, or high [55]. The assessment was performed using GRADE profiler (GRADEpro) software [55].

### Statistical Analysis

Study data were analyzed using Cochrane’s Review Manager (RevMan) 5.4.1 [56]. We used the immediate postintervention results in the analyses. To ensure consistent reporting, for outcomes reported as continuous variables, we calculated the standardized mean difference (SMD) and 95% CIs. If studies reported *P* values, we calculated the SD using RevMan’s inbuilt calculator [56]. If studies reported the median (IQR), we converted those values to means (SDs) following Luo et al [57] and Wan et al [58]. In studies reporting 2 or more groups, for example, studies with more than 1 intervention arm, we combined the groups before the analysis using the *Cochrane Handbook for Systematic Reviews of Interventions* guidelines [59]. Subsequently, we calculated the SMD and 95% CIs, as per the protocol. Effect size interpretation followed the Hedges’ g statistic and considered an intervention to have a small effect if the SMD was between 0.2 and 0.5, a moderate effect if it was between 0.5 and 0.8, and a large effect if it was 0.8 and above. For studies reporting dichotomous data, we calculated the relative risks (RRs) and associated 95% CIs across studies. For cRCTs, we extracted individual-level data, as reported in the primary studies. If relevant outcome data were missing from the primary studies, we contacted the authors to obtain information. We did not input any missing data. When possible, data were analyzed on an intention-to-treat basis.

We described our findings narratively using Miller’s classification of clinical competence [60] to classify the outcomes. When possible, we performed a meta-analysis using a random effects model. We used the *I*^2^ statistic to evaluate heterogeneity, with *I*^2^<25%, 25%-75%, and >75% representing low, moderate, and high heterogeneity, respectively [34].

We attempted to assess publication bias and perform subgroup analyses; however, the small number of studies in each category and the heterogeneity of data precluded these assessments.

## Results

### Characteristics of Included Studies

**Figure 1:**
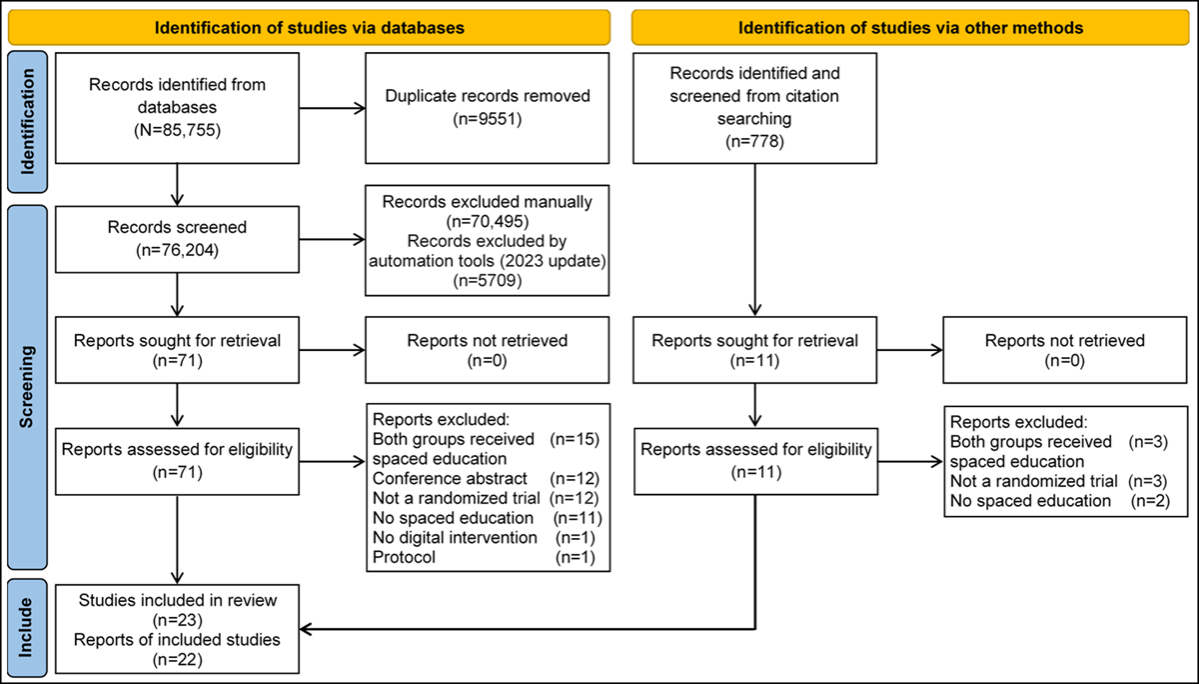
Study selection flowchart.

The search strategy yielded 85,755 publications, of which 23 (0.03%) studies, reported in 22 papers (0.03%), were included in the review. The study selection process was summarized using a PRISMA flow diagram (Figure 1) [35]. Multimedia Appendix 3 also presents a summary of the included studies, and Multimedia Appendix 4 lists the studies excluded after reviewing the full text.

Table 1 presents the characteristics of the included studies. Most studies (n=19, 83%) were RCTs. Participants were randomized into 2 groups, except in 1 (4%) study [61], which randomized participants into 3 groups. Most studies (n=19, 83%) were conducted in high-income countries [49,61-77], 3 (13%) studies were conducted in upper middle–income countries [78-80], and only 1 (4%) study was conducted in a lower middle–income country [81]. The studies included a total of 3371 participants. Study populations comprised postregistration health professionals (n=20, 87%) and preregistration medical [61,76] and nursing [79] students (n=3, 13%). Of the 23 studies, 9 (39%) [61,62,65,66,69,72,74,78,80] reported 1 and 7 (30%) [49,63,67,70,73,76,77] reported 2 primary outcomes. The most common outcome was knowledge (n=15, 65%) [49,62-68,72-75,77,81], followed by skills (n=9, 39%) [61,69,70,72,76,78-81], attitudes (n=8, 35%) [64,68,71-73,75,77,79], behavior change (n=8, 35%) [49,63,67,68,71,75,77], and satisfaction with the intervention (n=7, 31%) [64,70-72,75,76,81].

**Table 1:** Characteristics of the included studies.

Characteristics		Studies (N=23), n (%)
**Year of publication**		
	Before 2019	13 (57)
	After 2019	10 (43)
**Country**		
	United States	14 (61)
	Türkiye	2 (9)
	Australia	1 (4)
	Canada	1 (4)
	China	1 (4)
	France	1 (4)
	Nigeria	1 (4)
	Singapore	1 (4)
	United States and Canada	1 (4)
**Study design**		
	RCT^a^	19 (83)
	cRCT^b^	3 (13)
	Quasi-RCT	1 (4)
**Participants**		
	Students of medicine	2 (9)
	Students of nursing	1 (4)
	Medical and surgical specialty interns and residents (trainee physicians)	3 (13)
	Medical specialty residents (trainee physicians)	4 (17)
	Surgical specialty residents (trainee physicians)	5 (22)
	Practicing physicians	2 (9)
	Multidisciplinary primary care providers	6 (26)
**Clinical domain:** **medical specialties**		
	Internal medicine	2 (9)
	Oncology	1 (4)
	Pediatrics	2 (9)
	Primary care	5 (22)
**Clinical domain:** **surgical specialties**		
	General surgery	2 (9)
	Obstetrics and gynecology	2 (9)
	Orthopedics	2 (9)
	Urology	2 (9)
Clinical domain: medical and surgical specialties		2 (9)
**Intervention: spaced online education**		
	Via email	10 (43)
	Using proprietary software (QStream/Moodle)	4 (17)
	Via email with gamified elements	1 (4)
	Via mobile apps	1 (4)
	Via online games	1 (4)
**Intervention: s** **paced digital simulation training**		
	High-fidelity simulation	4 (17)
	VR^c^ simulation	1 (4)
	Enhanced with mobile learning	1 (4)
**Control: spaced online education**		
	No intervention	7 (30)
	Massed online education via email	5 (22)
	Online access to papers	2 (9)
	Massed digital education via app with no alerts/reminders	1 (4)
	Slideshow-based online program	1 (4)
	Standard curriculum	1 (4)
**Control: spaced** **digital simulation training**		
	Nonspaced high-fidelity simulation	3 (13)
	Nonspaced simulation	2 (9)
	No intervention	1 (4)
**Number of study outcomes**		
	1	9 (39)
	2	7 (30)
	3	5 (22)
	4	2 (9)
**Category of study outcomes**		
	Knowledge	15 (65)
	Skills	9 (39)
	Attitudes	8 (35)
	Satisfaction	7 (31)
	Behavior change	8 (35)
**Study quality**		
	Low risk of bias	11 (48)
	Unclear risk of bias	5 (22)
	High risk of bias	7 (30)

^a^RCT: randomized controlled trial.

^b^cRCT: cluster randomized controlled trial.

^c^VR: virtual reality.

### Characteristics of Interventions

Spaced online education was described in 17 (74%) studies. The interventions consisted of brief learning modules delivered at prespecified intervals using email (n=10, 59%) [49,63,65,66,69-72,77]; proprietary software, such as Qstream (n=3, 18%) [64,68,74] and Moodle (n=1, 6%) [62]; an online game (n=1, 6%) [75]; and a smartphone app (n=1, 6%) [73]. Typically, in 15 (88%) of the 17 studies, the learning modules presented a case study or a small learning unit, followed by 1 or more multiple-choice questions (MCQs) and the correct answer with an explanation to each question. Alternatively, 2 (12%) [69,70] of the 17 studies offered small learning units without a testing component. The information was repeated 2 or more times at predetermined intervals or according to participant responses in adaptive systems (n=10, 59%) [62,63,67,68,71-74,77]. The duration of the interventions varied from 1 week [75] to 16 months [73].

Additionally, 6 studies (26%) described spaced digital simulation interventions delivered using high-fidelity simulators (n=4, 67%) [76,78-80], a VR system (n=1, 17%) [61], or low-fidelity simulators enhanced with weekly SMS text messages (n=1, 17%) [81]. The duration of the interventions varied from 2 training sessions of 4 hours each delivered over 1 week (n=1, 17%) [79] to 4 training sessions delivered over 4 weeks (n=2, 33%) [76,80].

### Risk of Bias

**Figure 2:**
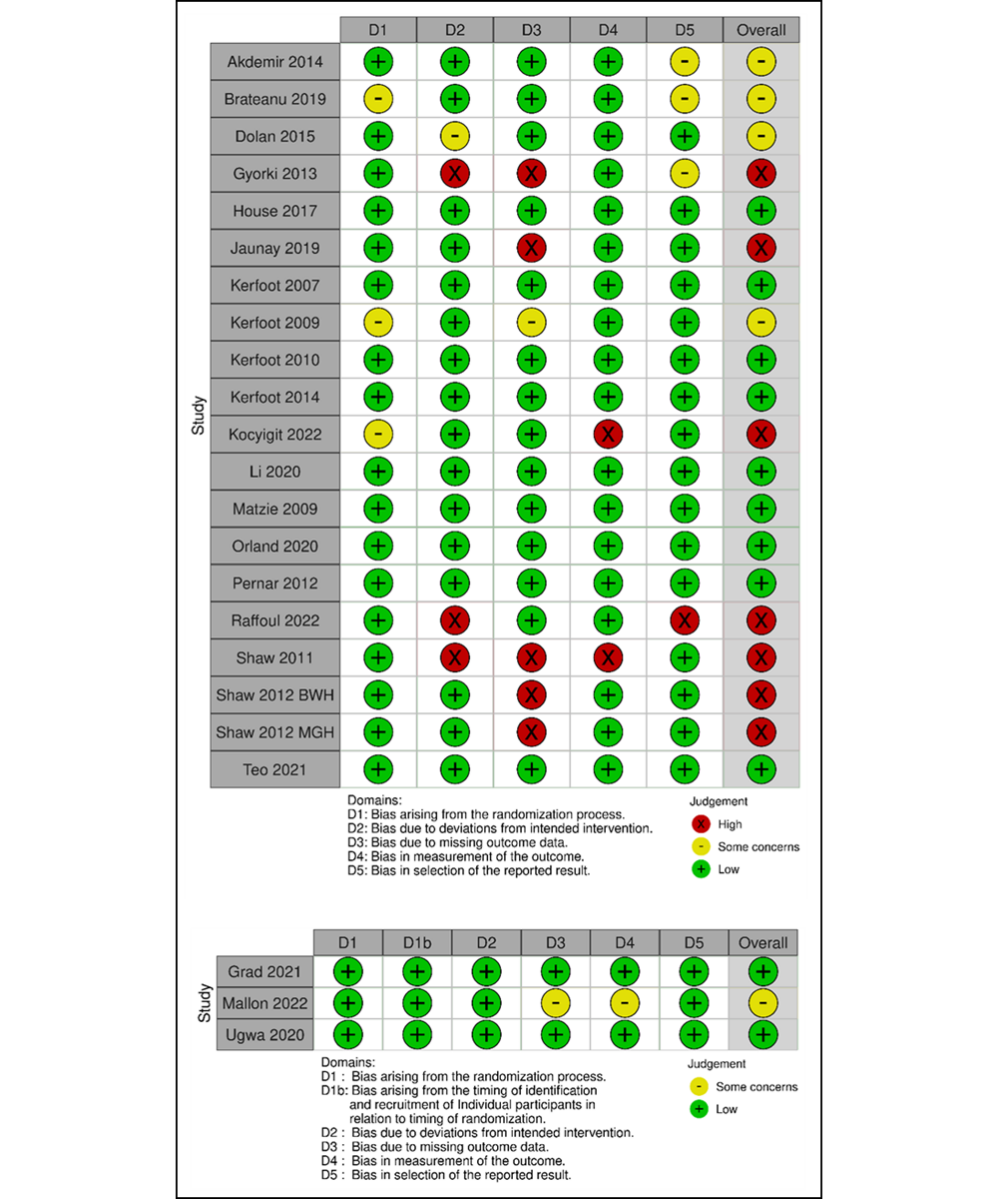
Risk-of-bias assessment for RCTs and cRCTs. cRCT: cluster randomized controlled trial; RCT: randomized controlled trial.

The risk of bias (Figure 2) was low in 9 (39%) studies [49,61,64,66,67,69,70,76,78], with some concerns in 4 (17%) studies [62,63,65,80], and high in 7 (30%) studies [71,72,74,75,77,79]. The most common source of bias was missing outcome data. The risk-of-bias assessment for 3 (13%) cRCTs [68,73,81] reflected some concerns in one study, while it was low in 2 studies.

### Primary Outcomes

#### Postintervention Knowledge

**Figure 3:**
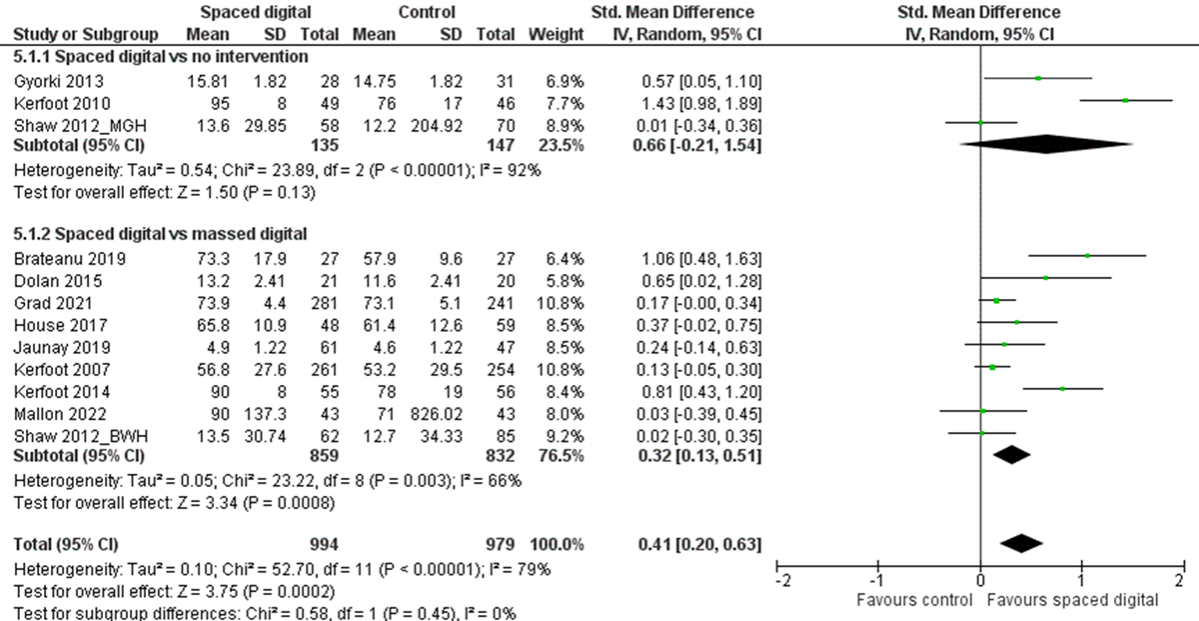
Forest plot for postintervention knowledge. Note: SD values for Dolan et al and Shaw et al (Brigham and Women’s Hospital and Massachusetts General Hospital) were derived from *P* values using RevMan’s inbuilt calculator.

Knowledge was reported by 15 (65%) studies (2924/3371, 86.74%, participants), including 14 (93%) studies on spaced online education [49,62-68,72-75,77] and 1 (7%) study on spaced digital simulation [81]. In total, 12 (80%) of 15 studies assessed knowledge using MCQs [49,62-68,72,74,81], of which 7 (47%) studies used validated questionnaires [49,63,65-68,81]. The other studies used a combination of MCQs and short-answer questions [75], the short-answer component of the College of Family Physicians of Canada [73], or true/false questions developed for the study [77]. Figure 3 [63,72] summarizes the pooled estimates for postintervention knowledge outcomes. Three studies presented knowledge retention data [64,65,81].

##### Spaced Online Education Versus No Intervention

Of 4 (27%) studies (499/2924, 17.07%, participants) [49,72,74,77], 3 (75%) studies (n=282, 56.5%, participants) [49,72,74] were included in the meta-analysis. The pooled estimate showed no significant difference in knowledge (SMD 0.66, 95% CI –0.21 to 1.54, *I*^2^=92%) between the intervention and control groups.

Another study (133/499, 26.7%, participants) [77], not included in the meta-analysis, randomized primary care providers (PCPs) to receive 2 months of spaced online education or no intervention after watching an educational video on eating disorders and reported no further improvement in knowledge in the intervention group.

##### Spaced Online Education Versus Massed Education

Of the 15 studies, 9 (60%) studies (1691/2924, 57.83%, participants) [62-64,66-68,72,73,75] comparing spaced online education with massed digital education were included in the meta-analysis. The pooled estimate showed a small effect size (SMD 0.32, 95% CI 0.13-0.51, *I*^2^=66%) favoring spaced online education.

##### Spaced Digital Simulation Versus Massed Simulation

Ugwa et al [81] compared low-dose onsite simulation enhanced with mobile mentoring with traditional massed simulation. Knowledge improved in the traditional arm immediately after completing the intervention.

##### Knowledge Retention

**Figure 4:**
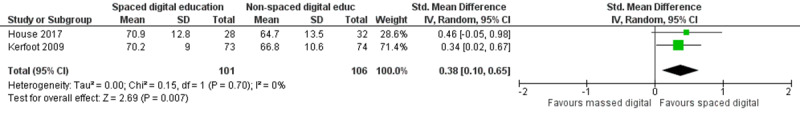
Forest plot for knowledge retention.

Of the 15 studies, 2 studies (313/2924, 10.7%, participants) [64,65] comparing spaced online education and massed education reported knowledge retention. House et al [64] reported a nonsignificant effect on knowledge retention at 3 months’ follow-up (SMD 0.46, 95% CI –0.05 to 0.98), while Kerfoot [65] reported a small effect size 2 years after the completion of a trial on spaced versus massed online education (SMD 0.34, 95% CI 0.02-0.67). The pooled estimate showed a small difference in knowledge retention favoring spaced online education over controls (SMD 0.38, 95% CI 0.10-0.65, *I*^2^=0%). Figure 4 presents the pooled estimates for knowledge retention.

Additionally, Ugwa et al [81] reported that knowledge retention scores at 3 and 12 months’ follow-up were similar in the spaced digital simulation and control arms (RR 1.02, 95% CI 0.91-1.13).

#### Postintervention Skills

A total of 9 (39%) studies (784/3371, 23.26%, participants) reported learners’ skills, including 3 (33%) studies on spaced online education [69,70,72] and 6 (67%) studies on spaced digital simulation [61,76,78-81]. Postintervention skills were measured using online surveys distributed among medical students to assess the faculty’s [70] or residents’ [69] teaching skills, anonymized video recordings [61,72], task completion reports obtained from the simulation instrument [76,78,80], the Objective Structured Clinical Examination (OSCE) [81], and self-reported questionnaires [79].

##### Spaced Online Education Versus No Intervention

**Figure 5:**
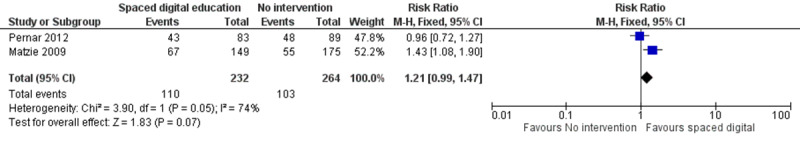
Forest plot for postintervention skills (spaced online education vs no intervention).

A total of 2 (22%) studies (84/784, 2.4%, participants) [69,70] compared the impact of spaced online education on improving teaching skills. Matzie et al [69] reported that residents receiving the intervention offered more effective feedback to medical students (RR 1.43, 95% CI 1.08-1.90), while Pernar et al [70] reported no difference in faculty’s feedback quality between intervention and control groups (RR 0.96, 95% CI 0.72-1.27). The pooled estimate for both studies reflected a nonsignificant effect of spaced online education on improving teaching skills (RR 1.21, 95% CI 0.99-1.47, *I*^2^=74%). Figure 5 presents the forest plot.

##### Spaced Online Education Versus Massed Education

Shaw et al [72] assessed interns’ compliance (Brigham and Women’s Hospital: 195/784, 24.9%, participants) with patient safety procedures while completing a central venous catheterization simulation, reporting a nonsignificant effect (SMD 0.34, 95% CI –0.05 to 0.73).

##### Spaced Digital Simulation Versus Massed Simulation

**Figure 6:**
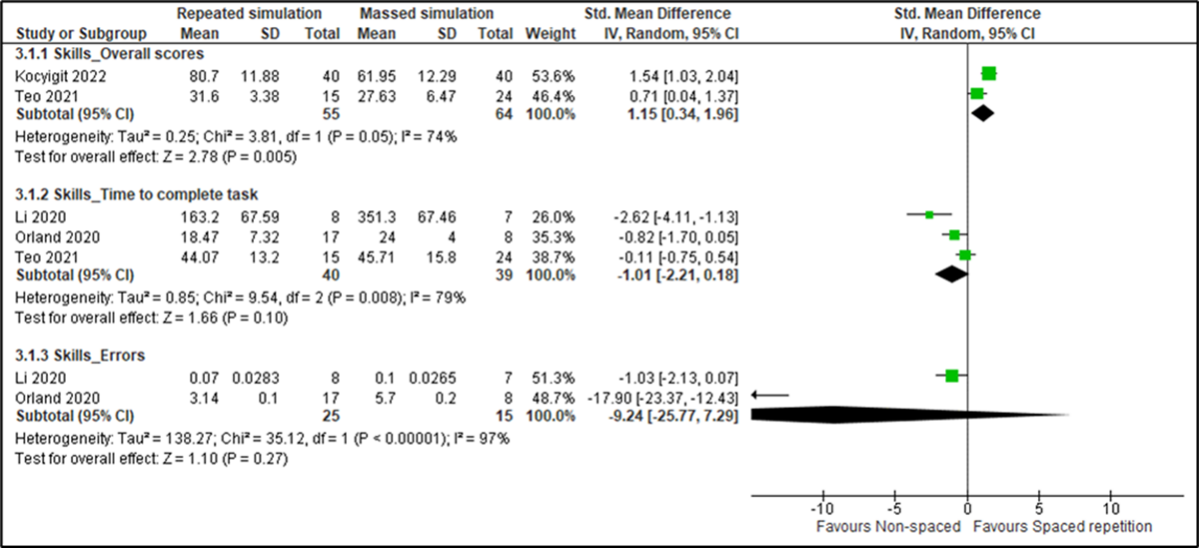
Forest plots for learners’ skill outcomes. Note: SD values for Li et al were derived from the SE using RevMan’s inbuilt calculator.

A total of 5 (56%) studies (483/784, 61.6%, participants) [61,76,78,79,81] compared spaced digital simulation with massed simulation (Figure 6 [11]). The interventions aimed to improve nursing students’ skills to manage an adult patient with chronic lymphocytic leukemia [79], residents’ arthroscopic skills [78], medical students’ intramedullary tibial nailing [61] and microsurgical suturing skills [76], and health workers’ maternal and newborn care skills [81]. In addition, 3 (60%) studies [76,79,81] reported skill improvement as overall scores. The pooled estimate of 2 (67%) studies [76,79] showed that spaced digital simulation is superior to massed simulation (SMD 1.24, 95% CI 0.84-1.64, *I*^2^=74%) in improving learners’ skills. However, Ugwa et al [81] reported a nonsignificant effect in learners’ skills using a spaced simulation intervention enhanced with mobile mentoring (RR 1.05, 95% CI 0.95-1.16) compared to controls.

In addition, 3 (60%) studies [61,76,78] reported itemized skill outcomes, including the time to complete a task, economy of movements, the number of errors, and the number of hints requested, with improved skills representing decreased values. Li et al [78] reported a decrease in task completion time and camera path length but no difference in the extent of cartilage injury in orthopedic residents training in arthroscopic skills using spaced high-fidelity simulation. Orland et al [61] reported a shorter completion time, decreased errors and number of requested hints, and a higher rate of completed tasks in medical students using spaced VR simulation with or without technique guidance compared to controls. Teo et al [76] reported a nonsignificant difference in the time to complete the test strip between students receiving spaced digital simulation and controls. The pooled estimates for the time to complete a task (3, 60%, studies) and the number of errors (2, 40%, studies) showed a nonsignificant difference between intervention and control groups.

##### Spaced Digital Simulation Versus No Intervention

Akdemir et al [80] (22/784, 2.8%, participants) described that a spaced digital simulation intervention to improve gynecology residents’ laparoscopic skills resulted in a significant reduction in the time to complete tasks (SMD –1.50, 95% CI –2.46 to –0.53) and the number of movements (SMD –1.96, 95% CI –3.01 to –0.91) and a nonsignificant change in the number of errors (SMD –0.26, 95% CI –1.10 to 0.58).

#### Postintervention Attitudes

A total of 8 (35%) studies (1539/3371, 45.65%, participants) reported attitudes, including confidence [64,68,71,72], comfort in screening [77], engagement with the intervention (time spent [75] and the number of completed cases [73]), self-efficacy [79], and anxiety [79].

##### Spaced Online Education Versus No Intervention

Raffoul et al [77] reported that PCPs in the intervention group were more comfortable screening for eating disorders. Shaw et al [71] showed that PCPs receiving spaced online education after a conference reported greater confidence in managing 4 conditions presented in the intervention.

##### Spaced Online Education Versus Massed Education

**Figure 7:**
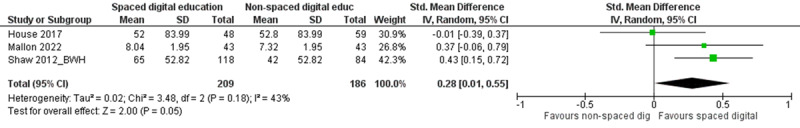
Forest plot for learners’ postintervention confidence (spaced online education vs massed education). Note: SD values for Shaw et al (Brigham and Women’s Hospital) were derived from *P* values, and SD values for House et al were derived from CIs using RevMan’s inbuilt calculator.

The pooled estimate of 3 (38%) studies showed improved confidence in managing clinical cases [64,68] and patient safety protocols [72] (SMD 0.28, 95% CI 0.01-0.55, *I*^2^=43%), as shown in Figure 7 [64,72].

Additionally, Jaunay et al [75] reported that general practitioners (GPs) who received spaced education via an online game spent 45-60 minutes in the intervention compared to 10-20 minutes in the control group. Grad et al [73] compared 2 versions of a study app presenting family medicine case studies and showed that adding reminders that enabled spaced learning resulted in residents completing more case studies, although the difference was nonsignificant.

##### Spaced Digital Simulation Versus Massed Simulation

Kocyigit and Karagozoglu [79] reported that spaced digital simulation improved nursing students’ self-efficacy (SMD 0.57, 95% CI 0.12-1.02), while there was no significant change in state anxiety (SMD 0.25, 95% CI –0.19 to 0.69) between the study groups.

##### Postintervention Satisfaction

A total of 7 (30%) studies (982/3371, 29.13%, participants) [64,70-72,75,76,81] reported satisfaction with spaced digital education. Satisfaction was assessed using a postintervention survey [75], Likert scale surveys [64,71,72,76], an email-based questionnaire [70], and a satisfaction questionnaire plus focus group discussion [81]. Satisfaction was measured only in the intervention group, except Teo et al [76], who administered the survey to both intervention and control groups and reported similar satisfaction (8.47/10 in spaced digital simulation vs 8.0/10 in massed simulation; *P*=.23). In general, participants considered spaced digital education engaging and enjoyable [72,75] and said that would like to engage in similar interventions [64,72,75] in the future. Participants also reported greater satisfaction in improved patient outcomes [81]. However, 1 (14%) study [70] reported that surgery faculty were not satisfied with an intervention consisting of weekly emails with tips to improve their teaching skills, because the content was vague and generic and it added to the burden of numerous daily emails.

#### Postintervention Behavior Change

**Figure 8:**
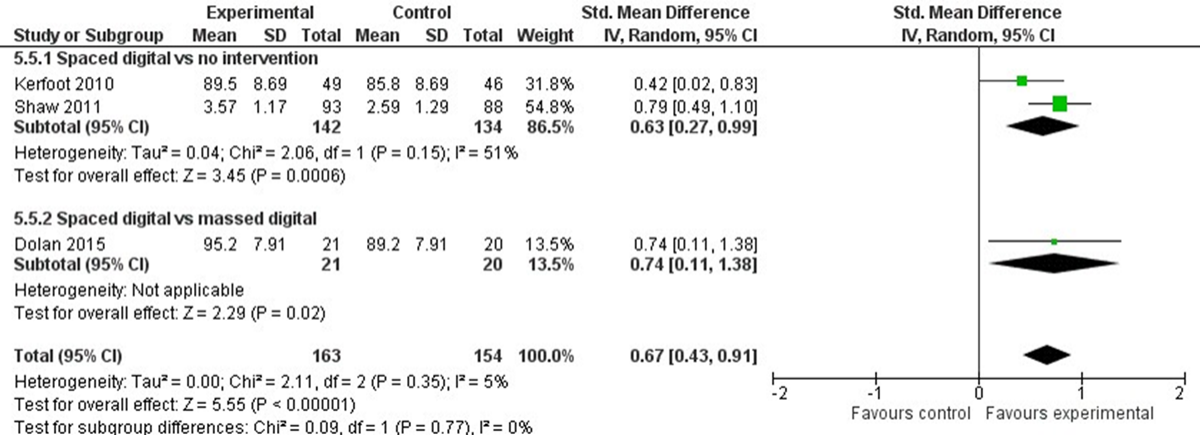
Forest plot for learners’ postintervention change in clinical behavior. Note: SD values for Dolan et al and Kerfoot et al were derived from *P* values using RevMan’s inbuilt calculator.

Of the 23 studies, 8 (35%) studies (858/3371, 25.45%, participants) reported a change in clinical behavior, including 7 (88%) studies on spaced online education [49,63,67,68,71,75,77] and 1 (12%) study on spaced digital simulation [79]. The behavior change data were obtained from clinical records [49,63,67,68] and self-reported questionnaires developed by the study authors [71,75,77,79]. Figure 8 [49,63] shows the meta-analyses results.

##### Spaced Online Education Versus No Intervention

The pooled estimate from 2 (25%) studies [49,71] showed that spaced online education promotes a change in clinicians’ behavior (SMD 0.63, 95% CI 0.27-0.99, *I*^2^=51%). Kerfoot et al [49] showed that spaced online education improved clinicians’ prostate cancer–screening behavior while receiving the intervention (SMD 0.42, 95% CI 0.02-0.83) and during the 72-week follow-up (SMD 0.53, 95% CI 0.12-0.94). Shaw et al [71] reported a change in PCPs’ clinical behavior for 4 common disorders (hypertension, diabetes, nonalcoholic steatohepatitis, and HIV) after receiving a spaced online education intervention (SMD 0.36, 95% CI 0.07-0.66 for hypertension to SMD 0.95, 95% CI 0.65-1.26 for HIV). Finally, Raffoul et al [77] reported that PCPs receiving spaced online education were more comfortable screening for eating disorders, although there was no difference between the intervention and control groups in self-reported screening or referral behavior.

##### Spaced Online Education Versus Massed Education

Dolan et al [63] reported that residents receiving spaced online education appropriately screened more patients for osteoporosis (SMD 0.74, 95% CI 0.11-1.38) and were more likely to treat patients with bisphosphonates (SMD 0.69, 95% CI 0.06-1.32). Jaunay et al [75] reported that GPs receiving an intervention involving a spaced online education game used the new knowledge to improve clinical practice more often (RR 1.45, 95% CI 1.07-1.96), compared to controls. Kerfoot et al [67] demonstrated improved patient outcomes (shorter time to blood pressure target) for PCPs receiving spaced online education (hazard ratio 1.043, 95% CI 1.007-1.081, *P*=.018). Finally, Mallon et al [68] reported that a spaced online constipation management program delivered to pediatric PCPs resulted in lower patient referrals to specialist pediatric gastroenterology clinics but no reduction in constipation-related visits to the emergency department or urgent care clinics.

##### Spaced Digital Simulation Versus Massed Simulation

Kocyigit and Karagozoglu [79] reported that nursing students’ medical error tendency significantly decreased after 2 spaced digital simulation sessions (80.7 vs 61.9 points, *P*=0, with higher values reflecting fewer medical errors).

## Discussion

### Principal Findings

In this systematic review, the first to include a meta-analysis, we found that spaced digital education is effective in improving knowledge, skills, and confidence and promoting change in clinical practice for pre- and postregistration health professionals. In particular, marked improvements were demonstrated in physicians’ adoption of evidence-based practices after engaging in spaced online learning and in students’ and residents’ surgical skills after engaging in spaced digital simulation interventions compared to active or inactive controls. The results for knowledge and behavior change, albeit reported in a smaller number of papers, showed that these improvements are maintained over time. Yet, our results should be interpreted with caution, given the substantial heterogeneity of included studies and the small number of studies populating the different spaced digital education categories.

Our systematic review categorized the spaced interventions into 2 distinct categories: spaced online education interventions repeatedly delivered small amounts of information while testing learners’ knowledge, while spaced digital simulation interventions provided practical, repeated, and spaced training sessions using high-fidelity simulators to improve learners’ skills. The studies included in this review evaluated 3 learning outcomes well aligned with the 4 levels of Miller’s classification of clinical competence: knowledge (“knows”), skills (“knows how,” “shows how”), and behavior change (“does”) [59]. Although measuring learners’ attitudes and satisfaction with the digital health education intervention is important and may facilitate learners’ engagement with the intervention, knowledge, skills, and behavior change are particularly important to determine the impact of the intervention. Knowledge or skills provide a direct evaluation of the intervention, and selecting one over the other will directly depend on the type of educational intervention content to be delivered. Alternatively, behavior change would more directly reflect the impact of the digital health education intervention on health professionals’ daily practice and the potential benefits to patients.

The findings reported in this review support the use of spaced digital education interventions in undergraduate and postgraduate health profession education. However, medical schools and providers of continuous professional development programs have yet to fully embrace this learning modality [82-84]. Simultaneously, students are increasingly turning to commercial learning platforms to extend their learning and to prepare for licensing examinations, such as the USMLE (United States Medical Licensing Examination) program [82,85]. These platforms often follow spaced education and active recall principles in the development of learning materials. About one-third of second-year medical students in the United States routinely use online materials not provided by their medical school to prepare for courses and particularly in preparation for the USMLE [86-88], creating a “parallel, medical student–driven curriculum” [86]. As digital health education platforms become more pervasive, it is critical that institutions providing undergraduate and continuous postgraduate development programs adapt their curriculum to incorporate evidence-based learning approaches to improve students’ learning outcomes, while increasing student satisfaction and acceptability of the educational content.

The studies included in this review described interventions with substantially diverse spacing intervals. However, none of the studies provided a rationale for this differing length. Previous studies from the educational research field have shown that the length of the spacing interval is directly related to the knowledge retention period, suggesting that the ideal interval would be around 10% of the test delay [23,89]. Furthermore, expanding the spacing interval appears to further improve retention [89]. Most of these studies have reported solely on knowledge, such as vocabulary recall [23]. Similar research in health profession education is lacking. Therefore, it is imperative that future research evaluate the role of the spacing effect on knowledge retention in health care professionals, as well as the impact of spaced digital education on other learning outcomes, such as skills or clinical behavior change.

Spaced online education interventions have been mostly delivered via email, which may not fully leverage more interactive, engaging features often included in other digital interventions. For example, a recent literature review [90] suggested that the use of game design elements in the development of digital health education interventions may increase user engagement and enhance collaboration. In addition, microlearning, an educational approach that consists of brief, focused lessons, is gaining acceptance as a professional development method in the corporate sector and industry [91], encouraging the development of gamified digital platforms customizable to user needs. However, these platforms do not appear to be widely used in health care settings, despite the potential benefits of including multimedia content, as well as reminders, leaderboards, or other gamified elements, to increase user engagement [92].

### Strengths and Limitations

There are several strengths of this systematic review. We followed gold-standard Cochrane guidelines, and we used a comprehensive search strategy developed by expert librarians that was previously used in a series of systematic reviews on the effectiveness of digital health education modalities in health profession education [5,12-14,17,18,36,38,40,42-47]. The search strategy was supplemented by forward and backward reference screening.

However, the review also has limitations. First, our search strategy was developed to retrieve clinical trials on digital health education interventions without including spaced education–specific search terms. However, to compensate for this limitation, we added a backward and forward citation search of all included studies. Second, the included studies showed substantial heterogeneity: they reported diverse outcomes and used diverse measurement instruments and reporting variables that limited further data analyses. Third, the low quality of the included studies, reflected in the majority of unclear- and high-risk-of-bias assessments, added uncertainty to the validity of our results.

### Future Research

Several aspects of spaced digital education for health care professionals warrant further research. First, the optimal spacing interval for spaced interventions needs to be defined, accounting for the type of learning outcome targeted by the intervention (eg, knowledge, skills, behavior change, satisfaction), the health care profession, the career stage (pre-registration students or postregistration professionals), and the intended retention period. Second, the potential impact of newer, more interactive platforms in influencing active engagement of participants, their compliance with the intervention, and the improvement in measured outcomes warrants further investigation. Third, more research is needed to identify the areas of learning where a spaced intervention may be more appropriate. Fourth, studies evaluating possible adverse events associated with interventions are needed as adverse events were not reported in any of the studies included in this review. In addition, cost-effectiveness studies are required to assess the feasibility of large-scale implementation of this learning modality.

### Conclusion

This systematic review showed that spaced digital education is effective in improving knowledge, skills, and clinical behavior change in pre- and postregistration health care professionals. The improvement of surgical skills using spaced digital simulation and clinical behavior change after spaced online education interventions are particularly noteworthy. These findings suggest that spaced digital education could be effectively added to undergraduate health profession education and continuous professional education programs for practicing professionals. For the successful implementation of spaced digital education interventions in the health professions, we suggest considering the use of newer, more interactive platforms to deliver the interventions. Future studies should evaluate areas of learning where the intervention will be most effective and assess the ideal length of the intervention and the spacing interval.

## Appendix

Multimedia Appendix 1 Medline search strategy.

Multimedia Appendix 2 Grading of Recommendations, Assessment, Development and Evaluation (GRADE)–based evaluation: summary of findings.

Multimedia Appendix 3 Characteristics of included studies.

Multimedia Appendix 4 Excluded studies.

Multimedia Appendix 5 PRISMA 2020 checklist.

## Abbreviations

cRCT: cluster randomized controlled trial
GRADE: Grading of Recommendations, Assessment, Development and Evaluation
MCQ: multiple-choice question
PCP: primary care provider
PRISMA: Preferred Reporting Items of Systematic Reviews and Meta-Analyses
RR: relative risk
SMD: standardized mean difference
USMLE: United States Medical Licensing Examination
VR: virtual reality

## Data Availability

The data sets supporting the conclusions of this paper are included within the paper and Multimedia Appendices.
