# Supplementary material for: Spaced Digital Education for Health Professionals: Systematic Review and Meta-Analysis
Source: J Med Internet Res. 2024 Oct 10;26:e57760. doi: 10.2196/57760 (PMC11502984; doi:10.2196/57760)
Supplement: Multimedia Appendix 2 [file jmir_v26i1e57760_app2.docx]

# Multimedia Appendix 2: GRADE Assessment – Summary of Findings Tables

## **Table 1**: Spaced online education compared to massed digital education for health professions education

| **Spaced online education compared to massed digital education for health professions education** | | | | | | |
| --- | --- | --- | --- | --- | --- | --- |
| **Patient or population:** Health professions education  **Setting:** Hospitals, outpatient clinics and universities  **Intervention:** Spaced online education  **Comparison:** Massed digital education | | | | | | |
| Outcomes | **Anticipated absolute effects^*^** (95% CI) | | Relative effect (95% CI) | № of participants (studies) | Certainty of the evidence (GRADE) | Comments |
|  | **Risk with massed digital** | **Risk with Spaced digital** |  |  |  |  |
| Knowledge | - | SMD **0.32 SD higher** (0.13 higher to 0.51 higher) | - | 1691 (9 RCTs) | ⨁⨁⨁◯ Moderate^a^ |  |
| Skills | - | SMD **0.34 higher** (0.05 lower to 0.73 higher) | - | 101 (1 RCT) | ⨁⨁◯◯ Low^a,c^ |  |
| Change in clinical behaviour | - | SMD **0.74 higher** (0.11 higher to 1.38 higher) | - | 41 (1 RCT) | ⨁⨁◯◯ Low^c^ |  |
| Satisfaction | - | SMD **0.31 higher** (0.03 higher to 0.59 higher) | - | 202 (1 RCT) | ⨁⨁◯◯ Low^a,d^ |  |
| Confidence | - | SMD **0.28 higher** (0.01 higher to 0.55 higher) | - | 395 (3 RCTs) | ⨁⨁⨁◯ Moderate^a^ |  |
| Knowledge retention | - | SMD **0.38 higher** (0.1 higher to 0.65 higher) | - | 207 (2 RCTs) | ⨁⨁◯◯ Low^a,e^ |  |
| ***The risk in the intervention group** (and its 95% confidence interval) is based on the assumed risk in the comparison group and the **relative effect** of the intervention (and its 95% CI). **CI:** confidence interval; **SMD:** standardised mean difference | | | | | | |
| **GRADE Working Group grades of evidence** **High certainty:** we are very confident that the true effect lies close to that of the estimate of the effect. **Moderate certainty:** we are moderately confident in the effect estimate: the true effect is likely to be close to the estimate of the effect, but there is a possibility that it is substantially different. **Low certainty:** our confidence in the effect estimate is limited: the true effect may be substantially different from the estimate of the effect. **Very low certainty:** we have very little confidence in the effect estimate: the true effect is likely to be substantially different from the estimate of effect. | | | | | | |

#### **Explanations**

a. Rated down by one level for study limitations: Risk of bias was rated as uncertain or high for most studies

b. Rated down by one level for inconsistency: the heterogeneity is high, there is a large variation in effect sizes and lack of overlap of CIs.

c. Rated down by two levels for imprecision: only 1 study, with a small sample size, was included in the outcome

d. Rated down by one level for imprecision: only 1 study was included in the outcome

e. Rated down by one level for imprecision: the number of pooled participants is smaller than the number of participants required in an appropriately powered clinical trial (optimal information size)

## **Table 2**: Spaced online education compared to no intervention for health professions education

| **Spaced online education compared to no intervention for health professions education** | | | | | | |
| --- | --- | --- | --- | --- | --- | --- |
| **Patient or population:** Health professions education  **Setting:** Hospitals, outpatient clinics and universities  **Intervention:** Spaced online education  **Comparison:** no intervention | | | | | | |
| Outcomes | **Anticipated absolute effects^*^** (95% CI) | | Relative effect (95% CI) | № of participants (studies) | Certainty of the evidence (GRADE) | Comments |
|  | **Risk with no intervention** | **Risk with Spaced digital** |  |  |  |  |
| Knowledge | - | SMD **0.66 higher** (0.21 lower to 1.54 higher) | - | 282 (3 RCTs) | ⨁◯◯◯ Very low^a,b,c^ |  |
| Skills - frequency of feedback | 390 per 1,000 | **472 per 1,000** (386 to 574) | **RR 1.21** (0.99 to 1.47) | 496 (2 RCTs) | ⨁⨁◯◯ Low^a,b^ |  |
| Behaviour | - | SMD **0.69 higher** (0.21 higher to 1.16 higher) | - | 276 (2 RCTs) | ⨁⨁◯◯ Low^b,c^ |  |
| ***The risk in the intervention group** (and its 95% confidence interval) is based on the assumed risk in the comparison group and the **relative effect** of the intervention (and its 95% CI). **CI:** confidence interval; **RR:** risk ratio; **SMD:** standardised mean difference | | | | | | |
| **GRADE Working Group grades of evidence** **High certainty:** we are very confident that the true effect lies close to that of the estimate of the effect. **Moderate certainty:** we are moderately confident in the effect estimate: the true effect is likely to be close to the estimate of the effect, but there is a possibility that it is substantially different. **Low certainty:** our confidence in the effect estimate is limited: the true effect may be substantially different from the estimate of the effect. **Very low certainty:** we have very little confidence in the effect estimate: the true effect is likely to be substantially different from the estimate of effect. | | | | | | |

#### **Explanations**

a. Rated down by one level for study limitations: Risk of bias was rated as uncertain or high for most studies

b. Rated down by one level for inconsistency: the heterogeneity is high

c. Rated down by one level for imprecision: the number of pooled participants is smaller than the number of participants required in an appropriately powered clinical trial (optimal information size)

## **Table 3**: Spaced digital simulation compared to massed simulation for health professions education

| **Spaced digital simulation compared to massed simulation for health professions education** | | | | | | |
| --- | --- | --- | --- | --- | --- | --- |
| **Patient or population:** Health professions education  **Setting:** Hospitals, outpatient clinics and universities  **Intervention:** Spaced digital simulation  **Comparison:** Massed simulation | | | | | | |
| Outcomes | **Anticipated absolute effects^*^** (95% CI) | | Relative effect (95% CI) | № of participants (studies) | Certainty of the evidence (GRADE) | Comments |
|  | **Risk with massed simulation** | **Risk with Spaced digital simulation** |  |  |  |  |
| Skills - Overall scores (SMD) | - | SMD **1.15 higher** (0.34 higher to 1.96 higher) | - | 119 (2 RCTs) | ⨁⨁◯◯ Low^a,b^ |  |
| Skills - Overall scores (RR) | 819 per 1,000 | **860 per 1,000** (778 to 950) | **RR 1.05** (0.95 to 1.16) | 299 (1 RCT) | ⨁⨁◯◯ Low^c,d^ |  |
| Skills - Time to complete task | - | SMD **1.01 lower** (2.21 lower to 0.18 higher) | - | 79 (3 RCTs) | ⨁⨁◯◯ Low^b,c^ |  |
| Skills - Errors | - | SMD **9.24 lower** (25.77 lower to 7.29 higher) | - | 40 (2 RCTs) | ⨁◯◯◯ Very low^b,d^ |  |
| Attitudes - Anxiety | - | SMD **0.25 higher** (0.19 lower to 0.69 higher) | - | 80 (1 RCT) | ⨁◯◯◯ Very low^a,c,e^ |  |
| Attitudes - Self-efficacy | - | SMD **0.57 higher** (0.12 higher to 1.02 higher) | - | 80 (1 RCT) | ⨁⨁◯◯ Low^a,e^ |  |
| Satisfaction | - | SMD **0.4 higher** (0.25 lower to 1.05 higher) | - | 39 (1 RCT) | ⨁⨁◯◯ Low^a,e^ |  |
| ***The risk in the intervention group** (and its 95% confidence interval) is based on the assumed risk in the comparison group and the **relative effect** of the intervention (and its 95% CI). **CI:** confidence interval; **RR:** risk ratio; **SMD:** standardised mean difference | | | | | | |
| **GRADE Working Group grades of evidence** **High certainty:** we are very confident that the true effect lies close to that of the estimate of the effect. **Moderate certainty:** we are moderately confident in the effect estimate: the true effect is likely to be close to the estimate of the effect, but there is a possibility that it is substantially different. **Low certainty:** our confidence in the effect estimate is limited: the true effect may be substantially different from the estimate of the effect. **Very low certainty:** we have very little confidence in the effect estimate: the true effect is likely to be substantially different from the estimate of effect. | | | | | | |

#### **Explanations**

a. Rated down by one level for study limitations: Risk of bias was rated as uncertain or high for most studies

b. Rated down by one level for imprecision: the number of pooled participants is smaller than the number of participants required in an appropriately powered clinical trial (optimal information size), there is high variability of effect sizes and lack of overlap of CIs

c. Rated down by one level for inconsistency: the heterogeneity is high, there is a large variation in effect sizes and/or lack of overlap of CIs.

d. Rated down by two levels for inconsistency: the heterogeneity is high, there is a large variation in effect sizes and/or lack of overlap of CIs.

e. Rated down by one level for imprecision: only 1 study, with a small sample size, was included in the outcome
