# Supplementary material for: Spaced Digital Education for Health Professionals: Systematic Review and Meta-Analysis
Source: J Med Internet Res. 2024 Oct 10;26:e57760. doi: 10.2196/57760 (PMC11502984; doi:10.2196/57760)
Supplement: Multimedia Appendix 3 [file jmir_v26i1e57760_app3.docx]

## Multimedia Appendix 3: Characteristics of included studies

| **Author, country, study design** | **Participants** | **Type of comparison** | **Intervention & Control groups** | **Measured outcomes** | | | | | **Effect size of interventions SMD [95% CI]** | **Interpretation of results** |
| --- | --- | --- | --- | --- | --- | --- | --- | --- | --- | --- |
|  |  |  |  | **Knowledge** | **Skills** | **Attitudes** | **Satisfaction** | **Behavior change** |  |  |
| Akdemir et al [1], Türkiye, RCT | **22** gynecology residents  *Int*: 11  *Control*: 11 | Repeated simulation vs no intervention | **Int**: High fidelity simulation  **Control**: No intervention | Not measured | Time to complete task, economy of movement, error scores | Not measured | Not measured | Not measured | **Skills:**  *Time to complete task*: -1.5 [-2.46, -0.53]  *Path length*:  -1.96 [-3.01, -0.91]  *Errors*:  -0.26 [-1.10, 0.58] | Large effect  Large effect  No significant effect |
| Brateanu et al [2], United States, RCT | **54** internal medicine residents  *Int*: 27  *Control*: 27 | Spaced digital vs massed digital | **Int**: Spaced digital education (PDFs + Moodle-delivered online flip cards)  **Control**: PDFs only | 40-item pre- and post-int medical knowledge assessment + 8-item independent post-int assessment | Not measured | Not measured | Not measured | Not measured | **Knowledge**:  1.06 [0.48, 1.63] | Large effect |
| Dolan et al [3], United States, RCT | **41** internal medicine residents  *Int*: 21  *Control*: 20 | Spaced digital vs massed digital | **Int**: Spaced digital education via email  **Control**: Massed digital education via email | 25-item MCQs | Not measured | Not measured | Not measured | Patient-level outcome data | **Knowledge**:  0.65 [0.02, 1.28]  **Behavior change**:  0.74 [0.11, 1.38] | Medium effect  Medium effect |
| Grad et al [4], Canada, cRCT | **522** family medicine residents  *Int*: 281  *Control*: 241 | Spaced digital vs massed digital | **Int:** Spaced digital education via app with preset alerts/ reminders  **Control:** Massed digital education via app with no alerts/ reminders | Participants’ score on the SAMP component of the 2019 certification examination of the CFPC | Not measured | Number of clinical cases completed by participants over 16-months of follow up | Not measured | Not measured | **Knowledge:**  0.17 [-0.00, 0.34]  **Attitudes:**  1.16 [0.81, 1.66] | Small effect  Large effect |

| **Author, country, study design** | **Participants** | **Type of comparison** | **Intervention & Control groups** | **Measured outcomes** | | | | | **Effect size of interventions SMD [95% CI]** | **Interpretation of results** |
| --- | --- | --- | --- | --- | --- | --- | --- | --- | --- | --- |
|  |  |  |  | **Knowledge** | **Skills** | **Attitudes** | **Satisfaction** | **Behavior change** |  |  |
| Gyorki et al [5], Australia, RCT | **97** residents of general surgery, and medical and radiation oncology  *Int*: 49  *Control*: 48 | Spaced digital vs no intervention | **Int**: Spaced digital education via Qstream  **Control**: No intervention | 22 questions developed by the research team | Not measured | Not measured | Not measured | Not measured | **Knowledge**:  0.57 [0.05, 1.09] | Not significant effect |
| House et al [6], United States, RCT | **107** emergency medicine and pediatric residents  *Int*: 48  *Control*: 59 | Spaced digital vs massed digital | **Int**: Standard curriculum + spaced digital education via Qstream  **Control**: Standard curriculum | 20-question MCQs obtained from the American Academy of Pediatrics | Not measured | Clinical confidence (5-point Likert scale) | Enjoyment and interest using the tool (5-point Likert scale) | Not measured | **Knowledge**:  0.37 [-0.02, 0.75]  **Attitudes**:  -0.01 [-0.39, 0.37] | Not significant effect  Not significant effect |
| Jaunay et al [7], France, RCT | **108** GPs  *Int*: 61  *Control*: 47 | Spaced digital vs massed digital | **Int**: Spaced digital education via online prototype video game (Hygie)  **Control**: Massed digital education via online article access | DQ-5: 5-item MCQ or free-text questions (pre-and post-test)  SQ-20: 20-item (DQ-5 + 15 questions) MCQ or free-text questions (post-test). | Not measured | Time spent playing by participants assigned to Hygie | 8-question satisfaction questionnaire completed at the end of the 1-week learning period | Self-reported use of the acquired knowledge in medical practice | **Knowledge:**  0.24 [-0.14, 0.62]  **Attitudes**:  *Time spent*:  *Int*: 45-60 min  *Control*: 10-20 min  **Satisfaction:**  87% participants Hygie useful for knowledge update  75% participants Hygie useful for CME credits  **Behavior change:**  RR 1.45 [1.07, 1.96] | Not significant effect |
| Kerfoot et al [8], United States and Canada, RCT | **515** urology residents  *Int***:** 261  *Control***:** 254 | Spaced digital vs massed digital | **Int:** Spaced digital education via email  **Control:** Massed digital education via email | Urology ISE | Not measured | Not measured | Not measured | Not measured | **Knowledge:**  0.13 [-0.05, 0.30] | Not significant effect |

| **Author, country, study design** | | **Participants** | **Type of comparison** | **Intervention & Control groups** | **Measured outcomes** | | | | | | | | | | **Effect size of interventions SMD [95% CI]** | | **Interpretation of results** | |
| --- | --- | --- | --- | --- | --- | --- | --- | --- | --- | --- | --- | --- | --- | --- | --- | --- | --- | --- |
|  |  |  |  |  | **Knowledge** | | **Skills** | | **Attitudes** | | **Satisfaction** | | **Behavior change** | |  |  |  |  |
| Kerfoot [9], United States, RCT | **206** urology residents  *Int*: 104  *Control*: 102 | Spaced digital vs massed digital | **Int**: Spaced digital education via email  **Control**: Massed digital education via email | | Retention after 2 years: MCQs (n=60) delivered to residents from Sep to Nov 2007 | | Not measured | | Not measured | | Not measured | | Not measured | | **Knowledge retention**:  0.34 [0.02, 0.67] | | Small effect | |
| Kerfoot et al [10], United States, RCT | **95** PCPs (physicians, nurses, and physician assistants)  *Int*: 49  *Control*: 46 | Spaced digital vs no intervention | **Int**: Spaced digital education via email  **Control**: No intervention | | 14 MCQs delivered via email | | Not measured | | Not measured | | Not measured | | Percentage of inappropriate PSA screening performed | | **Knowledge:**  1.43 [0.98, 1.89]  **Behavior change:**  -0.42 [-0.83, -0.01] | | Large effect  Small effect | |
| Kerfoot et al [11], United States, RCT | **111** PCPs (physicians, nurses, and physician assistants)  *Int*: 55  *Control*: 56 | Spaced digital vs massed digital | **Int:** Spaced digital education via email with game mechanics  **Control:** Massed digital education via email with online posting | | 24-question pre- and post-intervention test | | Not measured | | Not measured | | Not measured | | Time to BP target (<140/90 mm Hg) | | **Knowledge:**  0.81 [0.43, 1.20]  **Behavior change:**  HR: 1.043 [1.007, 1.081] p=0.018 | | Large effect | |
| Kocyigit and Karagozoglu [12], Türkiye, quasi-RCT | **80** nursing students  *Int*: 40  *Control*: 40 | Repeated simulation vs non-repeated simulation | **Int:** Simulation using high fidelity simulator  **Control:** No control group | | Not measured | | CLL Patient Scenario Skill Assessment and Medical Error Situation Evaluation Checklist | | Self-Efficacy Scale  State Anxiety Inventory | | Not measured | | Not measured | | **Skills:**  1.54 [1.03, 2.04]  **Attitudes:**  *Self-Efficacy*: 0.57 [0.12, 1.02]  *Anxiety*: 0.25  [-0.19, 0.69] | | Large effect  Moderate effect  Not significant effect | |

| **Author, country, study design** | | **Participants** | **Type of comparison** | **Intervention & Control groups** | **Measured outcomes** | | | | | **Effect size of interventions SMD [95% CI]** | **Interpretation of results** |
| --- | --- | --- | --- | --- | --- | --- | --- | --- | --- | --- | --- |
|  |  |  |  |  | **Knowledge** | **Skills** | **Attitudes** | **Satisfaction** | **Behavior change** |  |  |
| Li et al [13], China, RCT | **16** orthopedic residents  *Int*: 8  *Control*: 8 | Repeated simulation vs non-repeated simulation | **Int**: Repeated high-fidelity simulation  **Control**: Non-repeated high-fidelity simulation | Not measured | Time to complete task, economy of movement, injury | Not measured | Not measured | Not measured | **Skills**:  *Time to complete task*: -2.62 [-4.11, -1.13]  *Path length*:  -1.42 [-2.59, -0.25]  *Injury*: -1.41 [-2.58, -0.24] | Large effect  Large effect    Large effect |  |
| Mallon et al [14], United States, cRCT | **86** PCPs (pediatricians, nurses, and physician assistants)  *Int*: 43  *Control*: 43 | Spaced digital vs massed digital | **Int**: Spaced digital education via Qstream  **Control**: Massed digital education via email (PowerPoint slide set) | 11-item MCQs on constipation knowledge | Not measured | Confidence managing typical and difficult constipation cases.  Awareness and use of local and national management guidelines | Self-assessment survey | EmD/UC and GI clinic visits retrieved from EHR data | **Knowledge:**  0.03 [-0.39, 0.45]  **Attitudes:**  0.36 [-0.06, 0.79]  **Satisfaction:** PCPs satisfied with SE; 90% would like SE-based CMEs  **Behavior change:** *EmD/UC visits*: no change  *GI visits*: GI clinic visits: decreased for all patients and remained low for int group | Not significant effect  Small effect |  |
| Matzie et al [15], United States, RCT | **55** surgery residents  *Int*: 28  *Control*: 27 | Spaced digital vs no intervention | **Int**: Spaced digital education via email  **Control**: No intervention | Not measured | Frequency and quality of resident feedback by medical students | Not measured | Not measured | Not measured | **Skills:**  RR: 1.43 [1.08, 1.90] |  |  |

| **Author, country, study design** | | **Participants** | **Type of comparison** | **Intervention & Control groups** | **Measured outcomes** | | | | | **Effect size of interventions SMD [95% CI]** | **Interpretation of results** |
| --- | --- | --- | --- | --- | --- | --- | --- | --- | --- | --- | --- |
|  |  |  |  |  | **Knowledge** | **Skills** | **Attitudes** | **Satisfaction** | **Behavior change** |  |  |
| Orland et al [16], United States, RCT | **25** medical students  *Int*: 8 VR, 9 VR and technique guide  *Control*: 8 | Repeated simulation vs non-repeated simulation | **Int**: Repeated high-fidelity simulation with or without technique guide  **Control**: Non-repeated high-fidelity simulation | Not measured | Time to complete task, errors, hints | Not measured | Not measured | Not measured | **Skills**:  *Time to complete task*: -0.83 [-1.86, 0.21]  *Errors*: -14.95 [-20.99, -8.90]  *Hints*: -4.73 [-6.85, -2.60] | Not significant effect  Large effect  Large effect |  |
| Pernar et al [17], United States, RCT | **29** general surgery faculty  *Int*: 15  *Control*: 14 | Spaced digital vs no intervention | **Int**: Spaced digital education via email  **Control**: No intervention | Not measured | Medical students’ evaluations of faculty members | Not measured | Faculty perceptions of the usefulness of the program | Not measured | **Skills**:  RR: 0.96 [0.72, 1.27]  **Satisfaction**: faculty not satisfied with the program |  |  |
| Raffoul et al [18], United States, RCT | **133** PCPs (physicians and nurses)  *Int*: 65  *Control*: 68 | Spaced digital education vs no intervention | **Int**: Spaced digital education via email  **Control**: No intervention | 21 true-false questions | Not measured | Comfort in screening and making referrals for ED | Not measured | Survey on screening and referral of pediatric patients for ED in the preceding 2 months | **Knowledge:**  No significant difference in ED knowledge  **Attitudes**: SE significantly improved comfort in screening for BN (p < .01) and BED (p < .01)  **Behavior change**:  significantly greater use by PCPs of any specific screening tools for EDs in the past 2 months |  |  |

| **Author, country, study design** | | | **Participants** | | **Type of comparison** | | **Intervention & Control groups** | | **Measured outcomes** | | | | | | | | | | **Effect size of interventions SMD [95% CI]** | | **Interpretation of results** | |
| --- | --- | --- | --- | --- | --- | --- | --- | --- | --- | --- | --- | --- | --- | --- | --- | --- | --- | --- | --- | --- | --- | --- |
|  |  |  |  |  |  |  |  |  | **Knowledge** | | **Skills** | | **Attitudes** | | **Satisfaction** | | **Behavior change** | |  |  |  |  |
| Shaw et al [19], United States, RCT | **181** PCPs (physicians, osteopathic doctors, nurses, and physician assistants)  *Int*: 93  *Control*: 88 | | Spaced digital vs no intervention (waitlist) | | **Int**: Spaced digital education via email  **Control:** No intervention (waitlist) | | Not measured | | Not measured | | Self-reported  confidence (5-point Likert scale) | | Perceived effectiveness  Providers’ interest in receiving future  SE programs | | Self-reported behavior change (5-point Likert scale) | | **Behavior change:**  0.95 [0.65, 1.26] | | Large effect | |  |  |
| Shaw et al [20], United States, RCT | | | **147** incoming medical and surgical interns (BWH)  *Int*: 62  *Control*: 85 | | Spaced digital vs massed digital | | **Int**: Spaced digital education via email  **Control**: Massed digital education (slideshow-based online program) | | 15-item MCQs tested knowledge around the NPSGs | | Central line simulation | | 7-question online exit survey on confidence around NPSGs | | 7-question online exit survey on intervention acceptability | | Not measured | | **Knowledge**:  0.02 [-0.30, 0.35]  **Skills**:  0.34 [-0.05, 0.73]  **Attitudes**:  0.43 [0.15, 0.72]  **Satisfaction**:  0.31 [0.03, 0.59] | | Not significant effect  Not significant effect  Small effect  Small effect | |
| Shaw et al [20], United States, RCT | | | **174** incoming medical and surgical interns (MGH)  *Int*: 85  *Control*: 89 | | Spaced digital vs no intervention | | **Int**: Spaced digital education via email  **Control**: No intervention | | 15-item MCQs tested knowledge around the NPSGs | | Not measured | | Not measured | | Not measured | | Not measured | | **Knowledge**:  0.01 [-0.34, 0.36] | | Not significant effect | |
| Teo et al [21], Singapore, HIC | | | **39** medical students  *Int*: 15  *Control*: 24 | | Repeated simulation vs non-repeated simulation | | **Int**: Spaced simulation training  **Control**: Non-repeated simulation training | | Not measured | | Training platform associated software was used to assess sutures on latex strips of standard size | | Not measured | | Rank scale 1-10 | | Not measured | | **Skills**:  0.71 [0.04, 1.37]  **Satisfaction**:  0.40 [-0.25, 1.05] | | Moderate effect  Not significant effect | |

| **Author, country, study design** | **Participants** | **Type of comparison** | **Intervention & Control groups** | **Measured outcomes** | | | | | **Effect size of interventions SMD [95% CI]** | **Interpretation of results** |
| --- | --- | --- | --- | --- | --- | --- | --- | --- | --- | --- |
|  |  |  |  | **Knowledge** | **Skills** | **Attitudes** | **Satisfaction** | **Behavior change** |  |  |
| Ugwa et al [22], Nigeria, cRCT | **323** multidisciplinary health workers  *Int*: 184  *Control*: 139 | Repeated simulation vs non-repeated simulation | **Int**: Repeated simulation training  **Control**: Traditional non-repeated simulation training | MCQs | OSCEs | Not measured | Satisfaction (quantitative) survey & FGDs | Not measured | **Knowledge**:  -0.39 [-0.62, -0.16]  **Skills**:  1.36 [0.73, 2.55]  **Satisfaction**:  Participants overall satisfied with improvement in skills | Small effect  Large effect |

BED: Binge Eating Disorder; BN: Bulimia Nervosa; BP: Blood Pressure; BWH: Brigham and Women’s Hospital; CFPC: College of Family Physicians of Canada; CME: Continuing Medical Education; CLL: Chronic Lymphocytic Leukemia; cRCT: Cluster Randomized Controlled Trial; DQ-5: Dynamic Questionnaire-5; ED: Eating Disorders; EmD: Emergency Department; EHR: Electronic Health Record; FES: Fundamentals of Endoscopic Surgery; FGD: Focus Group Discussion; GAGES: Global Assessment of Gastrointestinal Endoscopic Skills; GP: General Practitioner; HR: Hazard Ratio; Int: Intervention; ISE: In-Service Examination; MCQ: Multiple Choice Question; MGH: Massachusetts General Hospital; NPSG: National Patient Safety Goal; OSCE: Objective Structured Clinical Examination; PCP: Primary Care Provider; PSA: Prostate-Specific Antigen; RCT: Randomized Controlled Trial; RR: Risk Ratio; SAMP: Short-answer Management Problem; SE: Spaced Education; SQ-20: Static Questionnaire-20; UC: Urgent Care

## References

1. Akdemir A, Zeybek B, Ergenoglu AM, Yeniel AO, Sendag F. Effect of spaced training with a box trainer on the acquisition and retention of basic laparoscopic skills. Int J Gynaecol Obstet. 2014;127(3):309-13. PMID: 25176414. doi: 10.1016/j.ijgo.2014.07.015.
2. Brateanu A, Strang TM, Garber A, Mani S, Spencer A, Spevak B, et al. Using an adaptive, self-directed web-based learning module to enhance residents' medical knowledge prior to a new clinical rotation. Med Sci Educ. 2019;29(3):779-86. PMID: 34457542. doi: 10.1007/s40670-019-00772-8.
3. Dolan BM, Yialamas MA, McMahon GT. A randomized educational intervention trial to determine the effect of online education on the quality of resident-delivered care. J Grad Med Educ. 2015;7(3):376-81. PMID: 26457142. doi: 10.4300/JGME-D-14-00571.1.
4. Grad R, Leger D, Kaczorowski J, Schuster T, Adler S, Aman M, et al. Does spaced education improve clinical knowledge among Family Medicine residents? A cluster randomized controlled trial. Adv Health Sci Educ Theory Pract. 2021;26(3):771-83. PMID: 33389233. doi: 10.1007/s10459-020-10020-z.
5. Gyorki DE, Shaw T, Nicholson J, Baker C, Pitcher M, Skandarajah A, et al. Improving the impact of didactic resident training with online spaced education. ANZ J Surg. 2013;83(6):477-80. PMID: 23617607. doi: 10.1111/ans.12166.
6. House H, Monuteaux MC, Nagler J. A randomized educational interventional trial of spaced education during a pediatric rotation. AEM Educ Training. 2017;1(2):151-7. PMID: 30051026. doi: 10.1002/aet2.10025.
7. Jaunay LB, Zerr P, Peguin L, Renouard L, Ivanoff AS, Picard H, et al. Development and evaluation of a new serious game for continuing medical education of general practitioners (Hygie): double-blinded randomized controlled trial. J Med Internet Res. 2019;21(11):e12669. PMID: 31746775. doi: 10.2196/12669.
8. Kerfoot BP, Baker HE, Koch MO, Connelly D, Joseph DB, Ritchey ML. Randomized, controlled trial of spaced education to urology residents in the United States and Canada. J Urol. 2007;177(4):1481-7. PMID: 17382760. doi: 10.1016/j.juro.2006.11.074.
9. Kerfoot BP. Learning benefits of on-line spaced education persist for 2 years. J Urol. 2009;181(6):2671-3. PMID: 19375095. doi: 10.1016/j.juro.2009.02.024.
10. Kerfoot BP, Lawler EV, Sokolovskaya G, Gagnon D, Conlin PR. Durable improvements in prostate cancer screening from online spaced education a randomized controlled trial. Am J Prev Med. 2010;39(5):472-8. PMID: 20965387. doi: 10.1016/j.amepre.2010.07.016.
11. Kerfoot BP, Turchin A, Breydo E, Gagnon D, Conlin PR. An online spaced-education game among clinicians improves their patients' time to blood pressure control: a randomized controlled trial. Circ Cardiovasc Qual Outcomes. 2014;7(3):468-74. PMID: 24847084. doi: 10.1161/CIRCOUTCOMES.113.000814.
12. Kocyigit H, Karagozoglu S. Effects of scenario-based high fidelity and repeated simulation methods on the medical error tendency, self-efficacy and state anxiety levels of nursing students. Int J Caring Sci. 2022;15(2):1168-77.
13. Li W, Zhang KJ, Yao S, Xie X, Han W, Xiong WB, Tian J. Simulation-based arthroscopic skills using a spaced retraining schedule reduces short-term task completion time and camera path length. Arthroscopy. 2020;36(11):2866-72. PMID: 32502713. doi: 10.1016/j.arthro.2020.05.040.
14. Mallon D, Fei L, Farrell M, Anderson JB, Klein M. Randomized Controlled Trial of Interactive Spaced Education to Support Constipation Management by Pediatric Primary Care Providers. J Pediatr Gastroenterol Nutr. 2022;74(5):568-74. PMID: 35149642. doi: 10.1097/MPG.0000000000003405.
15. Matzie KA, Kerfoot BP, Hafler JP, Breen EM. Spaced education improves the feedback that surgical residents give to medical students: a randomized trial. Am J Surg. 2009;197(2):252-7. PMID: 18722585. doi: 10.1016/j.amjsurg.2008.01.025.
16. Orland MD, Patetta MJ, Wieser M, Kayupov E, Gonzalez MH. Does Virtual Reality Improve Procedural Completion and Accuracy in an Intramedullary Tibial Nail Procedure? A Randomized Control Trial. Clin Orthop Relat Res. 2020;478(9):2170-7. PMID: 32769533. doi: 10.1097/CORR.0000000000001362.
17. Pernar LI, Beleniski F, Rosen H, Lipsitz S, Hafler J, Breen E. Spaced education faculty development may not improve faculty teaching performance ratings in a surgery department. J Surg Educ. 2012;69(1):52-7. PMID: 22208833. doi: 10.1016/j.jsurg.2011.06.013.
18. Raffoul A, Vitagliano JA, Sarda V, Chan C, Chwa C, Ferreira KB, et al. Evaluation of a one-hour asynchronous video training for eating disorder screening and referral in U.S. pediatric primary care: a pilot study. Int J Eat Disord. 2022;55(9):1245-51. PMID: 35781822. doi: 10.1002/eat.23766.
19. Shaw T, Long A, Chopra S, Kerfoot BP. Impact on clinical behavior of face-to-face continuing medical education blended with online spaced education: a randomized controlled trial. J Contin Educ Health Prof. 2011;31(2):103-8. PMID: 21671276. doi: 10.1002/chp.20113.
20. Shaw TJ, Pernar LI, Peyre SE, Helfrick JF, Vogelgesang KR, Graydon-Baker E, et al. Impact of online education on intern behaviour around joint commission national patient safety goals: a randomised trial. BMJ Qual Saf. 2012;21(10):819-25. PMID: 22706930. doi: 10.1136/bmjqs-2011-000702.
21. Teo WZW, Dong XK, Yusoff S, Das De S, Chong AKS. Randomized controlled trial comparing the effectiveness of mass and spaced learning in microsurgical procedures using computer aided assessment. Sci Rep. 2021;11(1). PMID: 33531563. doi: 10.1038/s41598-021-82419-6.
22. Ugwa E, Kabue M, Otolorin E, Yenokyan G, Oniyire A, Orji B, et al. Simulation-based low-dose, high-frequency plus mobile mentoring versus traditional group-based trainings among health workers on day of birth care in Nigeria; a cluster randomized controlled trial. BMC Health Serv Res. 2020;20(1):586. PMID: 32590979. doi: 10.1186/s12913-020-05450-9.
