# Supplementary material for: Spaced Digital Education for Health Professionals: Systematic Review and Meta-Analysis
Source: J Med Internet Res. 2024 Oct 10;26:e57760. doi: 10.2196/57760 (PMC11502984; doi:10.2196/57760)
Supplement: Multimedia Appendix 4 [file jmir_v26i1e57760_app4.docx]

## Multimedia Appendix 4: Excluded studies

| **Reasons for exclusion** | **Study ID** | **Study DOI/Citation** |
| --- | --- | --- |
| Both groups received spaced education | Bjerrum 2016 | [10.3402/meo.v21.30517](https://doi.org/10.3402/meo.v21.30517) |
|  | Çan 2022 | [10.1186/s12909-022-03564-8](https://doi.org/10.1186/s12909-022-03564-8) |
|  | Frithioff 2022 | [10.1017/S0022215121003352](https://doi.org/10.1017/s0022215121003352) |
|  | Kerfoot 2007c | [10.1111/j.1365-2929.2006.02644.x](https://doi.org/10.1111/j.1365-2929.2006.02644.x) |
|  | Kerfoot, 2008 | [10.1007/s11606-008-0533-0](https://doi.org/10.1007/s11606-008-0533-0) |
|  | Kerfoot 2008a | [10.1016/j.juro.2008.01.126](https://doi.org/10.1016/j.juro.2008.01.126) |
|  | Kerfoot 2008b | [10.1007/s11606-008-0533-0](https://doi.org/10.1007/s11606-008-0533-0) |
|  | Kerfoot 2009b | [10.1016/j.amjsurg.2007.10.026](https://doi.org/10.1016/j.amjsurg.2007.10.026) |
|  | Kerfoot 2009d | [10.1097/SLA.0b013e31819f6db8](https://doi.org/10.1097/sla.0b013e31819f6db8) |
|  | Kerfoot 2010a | [10.1016/j.juro.2009.10.005](https://doi.org/10.1016/j.juro.2009.10.005) |
|  | Kerfoot 2010b | [10.1016/j.jamcollsurg.2010.04.023](https://doi.org/10.1016/j.jamcollsurg.2010.04.023) |
|  | Kerfoot 2011 | [10.1097/ACM.0b013e3182087bef](https://doi.org/10.1097/acm.0b013e3182087bef) |
|  | Kerfoot 2012 | [10.1097/SLA.0b013e31825b3912](https://doi.org/10.1097/sla.0b013e31825b3912) |
|  | Kun 2019 | [10.1016/j.jsurg.2018.08.017](https://doi.org/10.1016/j.jsurg.2018.08.017) |
|  | Marks 2020 | [10.1111/pedi.13010](https://doi.org/10.1111/pedi.13010) |
|  | Shenoi 2016 | [10.1177/2158244016653167](https://doi.org/10.1177/2158244016653167) |
|  | Taveira-Gomes 2015 | [10.1186/s12909-014-0275-0](https://doi.org/10.1186/s12909-014-0275-0) |
|  | Verdaasdonk, 2007 | [10.1007/s00464-005-0852-8](https://doi.org/10.1007/s00464-005-0852-8) |
| Conference abstract | Artinian 2017 | [10.1016/j.acap.2017.04.073](https://doi.org/10.1016/j.acap.2017.04.073) |
|  | Brateanu 2015 | [10.1007/s11606-015-3271-0](https://doi.org/10.1007/s11606-015-3271-0) |
|  | Dolan 2012 | [10.1007/s11606-012-2038-0](https://doi.org/10.1007/s11606-012-2038-0) |
|  | Kerfoot 2009c | [10.1038/modpathol.2008.214](https://doi.org/10.1038/modpathol.2008.214) |
|  | Kerfoot 2009e | [10.1016/S0022-5347(09)60548-6](https://doi.org/10.1016/S0022-5347(09)60548-6) |
|  | Klein 2017 | [10.1016/S0735-1097(17)35914-4](https://doi.org/10.1016/S0735-1097(17)35914-4) |
|  | Reed 2014 | [10.1111/acem.12365](https://doi.org/10.1111/acem.12365) |
|  | Sampson 2017 | [10.1097/01.AOG.0000471054.92856.31](https://journals.lww.com/greenjournal/abstract/2015/10001/interactive_spaced_education_as_an_adjunct_to.27.aspx) |
|  | Shaw 2011 | [10.1111/j.1743-7563.2011.01479.x](https://doi.org/10.1111/j.1743-7563.2011.01479.x) |
|  | Shenoi 2013 | [10.1111/acem.12115](https://doi.org/10.1111/acem.12115) |
|  | Von Roenn 2018 | [10.1177/0269216318769196](https://doi.org/10.1177/0269216318769196) |
|  | Walter 2014 | [10.1007/s11606-014-2834-9](https://doi.org/10.1007/s11606-014-2834-9) |
| Not a randomized trial | Ahmad 2023 | [10.46903/gjms/21.02.1233](https://doi.org/10.46903/gjms/21.02.1233) |
|  | Andersen 2015 | [10.1001/jamaoto.2015.1563](https://doi.org/10.1001/jamaoto.2015.1563) |
|  | Andersen 2016 | [10.1002/lary.25449](https://doi.org/10.1002/lary.25449) |
|  | Andersen 2016a | [10.1001/jamaoto.2016.0454](https://doi.org/10.1001/jamaoto.2016.0454) |
|  | Arain 2022 | [10.4103/jehp.jehp_825_21](https://doi.org/10.4103/jehp.jehp_825_21) |
|  | Barsoumian 2018 | [10.1093/milmed/usx020](https://doi.org/10.1093/milmed/usx020) |
|  | Boespflug 2015 | [10.1001/jamadermatol.2015.0214](http://dx.doi.org/10.1001/jamadermatol.2015.0214) |
|  | Gooding 2017 | [10.1016/j.jadohealth.2016.12.002](https://doi.org/10.1016/j.jadohealth.2016.12.002) |
|  | Matos 2017 | [10.1371/journal.pone.0181418](https://doi.org/10.1371/journal.pone.0181418) |
|  | McEvoy 2021 | [10.1097/ACM.0000000000003969](https://dx.doi.org/10.1097/ACM.0000000000003969) |
|  | Menon 2020 | [10.1186/1472-6920-13-45](https://doi.org/10.1186/1472-6920-13-45) |
|  | Recker 2022 | [10.1007/s00404-022-06656-4](https://doi.org/10.1007/s00404-022-06656-4) |
|  | Robertson 2023 | [10.3171/2023.1.JNS222651](https://doi.org/10.3171/2023.1.jns222651) |
|  | Tshibwabwa 2017 | [10.4103/jcis.JCIS_1_17](https://doi.org/10.4103/jcis.jcis_1_17) |
|  | Zheng 2022 | [10.1002/jdd.13066](https://doi.org/10.1002/jdd.13066) |
| No spaced education | Artinian 2020 | [10.4103/jehp.jehp_825_21](https://doi.org/10.4103/jehp.jehp_825_21) |
|  | Boettcher 2020 | [10.1055/s-0039-1681022](https://doi.org/10.1055/s-0039-1681022) |
|  | Boettcher 2021 | [10.1055/s-0040-1721041](https://doi.org/10.1055/s-0040-1721041) |
|  | Cassidy 2022 | [10.1007/s00464-021-08958-1](https://doi.org/10.1007/s00464-021-08958-1) |
|  | Elrod 2022 | [10.1055/s-0041-1741542](https://doi-org.libproxy1.nus.edu.sg/10.1055/s-0041-1741542) |
|  | Klein 2020 | [10.15766/mep_2374-8265.10927](https://doi.org/10.15766/mep_2374-8265.10927) |
|  | Kohli 2019 | Dent Res J (Isfahan). 2019 Sep-Oct; 16(5): 289–297 |
|  | Lammers 2021 | [10.1080/10903127.2021.1916140](https://doi.org/10.1080/10903127.2021.1916140) |
|  | Maier 2013 | [10.1186/1472-6920-13-45](https://doi.org/10.1186/1472-6920-13-45) |
|  | Nkenke 2012 | [10.1186/1472-6920-12-32](https://doi.org/10.1186/1472-6920-12-32) |
|  | Pitt 2022 | [10.1371/journal.pone.0279296](https://doi.org/10.1371/journal.pone.0279296) |
|  | Rustici 2020 | [10.1002/aet2.10366](https://doi.org/10.1002/aet2.10366) |
|  | Zarshenas 2022 | [10.1186/s12909-022-03726-8](https://doi.org/10.1186/s12909-022-03726-8) |
| No digital intervention | Joosten 2022 | [10.1007/s00464-022-09277-9](https://doi.org/10.1007/s00464-022-09277-9) |
| Protocol | Ugwa 2018 | [10.1186/s12913-018-3405-2](https://doi.org/10.1186/s12913-018-3405-2) |
